# Refining estimation techniques for the Two-Sided Power Distribution: A data-sensitive perspective

**DOI:** 10.1371/journal.pone.0340387

**Published:** 2026-01-23

**Authors:** Yunus Güral

**Affiliations:** Department of Statistics, Faculty of Science, Firat University, Elazığ, Türkiye; SUNY Upstate Medical University Hospital, UNITED STATES OF AMERICA

## Abstract

This study proposes a novel method aimed at achieving more reliable parameter estimates for the Two-Sided Power Distribution (TSPD), particularly under small-sample conditions. The proposed approach enhances the flexibility and data sensitivity of the distribution by redefining it as a convex combination of two independent uniform components. A new estimation formula is introduced for the probability Pr{Y<X}, which holds critical importance in fields such as system reliability and stress–strength modeling. Compared to classical theoretical expressions, this new formulation produces more accurate and stable results in small-sample settings. Simulation studies and real-data applications demonstrate that the proposed method reduces estimation error and yields values closer to empirical observations. Furthermore, the method provides a balanced and computationally feasible alternative to conventional techniques such as maximum likelihood estimation. The results reveal that the proposed approach offers a significant advantage for the reliable computation of key performance metrics such as reliability probabilities and related measures.

## 1. Introduction

In statistical modeling, the flexibility of probability distributions is one of the key determinants of model success, both in theoretical and practical contexts. A distribution’s flexibility refers to its ability to accommodate structural features of observed data—such as asymmetry, kurtosis, and modal behavior—through its parameters, and to allow stable and computationally feasible estimation of these parameters. Especially in applications involving limited sample sizes, the choice of distribution can significantly impact the reliability of the model and the generalizability of its results [1,2]. In such cases, distributions with compact support and low sensitivity to outliers are typically preferred.

In this context, the Two-Sided Power Distribution (TSPD) stands out as a flexible parametric model defined on the interval [0,1]. It is capable of producing asymmetric density structures centered around a mode point. The distribution was first introduced by Van Dorp and Kotz (2002) and has since been studied extensively in fields such as reliability, risk analysis, and financial modeling [3]. By going beyond classical unimodal structures, TSPD is particularly well-suited to capturing complex system behaviors that follow multi-phase patterns, such as initiation, steady-state, and deterioration [4,5].

This flexibility, however, also brings computational challenges. Classical estimation methods like Maximum Likelihood Estimation (MLE) often produce unstable or biased results in small-sample conditions because they rely on observed order statistics [6]. This makes it difficult to accurately estimate key performance metrics such as the probability Pr{Y<X}, which compares two independent random variables. Although analytical formulas for this probability exist under various distributions [7–9], they are mostly valid when samples are large and may differ from empirical results in small samples.

To overcome these challenges, several alternative methods have been proposed in the literature. For instance, Kotz and Seier (2008) introduced bootstrap-based corrections [10], while Lemonte (2024) proposed flexible beta-like models to improve estimation stability [2]. Nevertheless, such approaches are often computationally intensive or fall short in addressing the structural flexibility required by the underlying distribution.

In this study, a novel parametric formulation for the TSPD is introduced. This formulation redefines the probability density function as a convex combination of its two piecewise normalized components, corresponding to the intervals [0,θ] and [θ,1]. As a result, the model retains its asymmetric characteristics while offering enhanced continuity and optimization advantages in parameter estimation. Furthermore, the proposed approach simplifies the computation of reliability-related probabilities such as Pr{Y<X}, and increases sensitivity to small-sample dynamics. Similar to the stratified integration strategy proposed by Gökdere and Gürcan (2015), the density structure in this study is partitioned into nine subregions, enabling a more flexible and accurate estimation framework [11].

Accordingly, the main contributions of this study can be summarized as follows:

- The TSPD is reformulated as a convex combination of two uniform components.
- A simplified, computable, and small-sample-sensitive formulation is introduced for the probability Pr{Y<X}.
- Parameter estimation is conducted via numerical optimization over a continuous parameter space, overcoming the limitations of traditional MLE.
- The proposed method is validated through simulation studies and real-data applications, demonstrating strong empirical performance.
- Key challenges in the literature—such as data sensitivity, stability in small samples, and computational feasibility—are comprehensively addressed within this framework.

## 2. Materials and methods

### 2.1 Structure of the distribution and convex representation

The Two-Sided Power Distribution (TSPD) is a flexible probability distribution designed to model data constrained within a bounded interval, typically [0, 1]. Its structure divides the domain into two distinct regions, left and right, around a central breakpoint, allowing for asymmetric behaviors. This feature makes TSPD suitable for modeling systems with nonuniform density behavior, particularly in small-sample settings. The classical form of the TSPD probability density function (PDF) is given as,


$$f(x;\alpha ,\theta )=\left\{ \;\;\;\;\begin{array}{c} \begin{array}{cc} \alpha {\left(\frac{x}{\theta }\right)}^{\alpha -\text{1}}, & \text{0}\leq x\leq \theta \end{array}\\ \begin{array}{cc} \alpha {\left(\frac{\text{1}-x}{\text{1}-\theta }\right)}^{\alpha -\text{1}}, & \theta <x\leq \text{1} \end{array} \end{array}\right.\;$$ (1)

where α>0 is a shape (power) parameter, and θ∈[0, 1] is the structural breakpoint of the distribution. When α=1, the distribution reduces to the uniform; when α>1, the density increases toward the center; when  α<1, the density increases toward the edges [3].

Based on the classical PDF in Equation (1), each piece can be normalized on its respective interval, yielding the component densities


$$f_{\text{1}}(x;\alpha ,\theta )=\begin{array}{cc} \frac{\alpha }{\theta }{\left(\frac{x}{\theta }\right)}^{\alpha -\text{1}}, & \text{0}\leq x\leq \theta \end{array}$$ (2)

$$f_{\text{2}}(x;\alpha ,\theta )=\begin{array}{cc} \frac{\alpha }{\text{1}-\theta }{\left(\frac{\text{1}-x}{\text{1}-\theta }\right)}^{\alpha -\text{1}}, & \theta <x\leq \text{1} \end{array}$$ (3)

This decomposition highlights that $f_{\text{1}}(x)$ is defined over x increasing from 0 to θ, while $f_{\text{2}}(x)$ is defined over x decreasing from 1 to θ. The formulations in (2) and (3) can be written in the form of a convex combination as follows,


$$f(x)=\text{w}\;f_{\text{1}}(x)+(\text{1}-\text{w})\;f_{\text{2}}(x)$$ (4)

where w=Pr{X≤θ}. Equation (4) maintains the validity of the probability distribution while allowing smoother and more data-sensitive transitions between the two subregions.

### 2.2 Maximum Likelihood (MLE)

Maximum Likelihood Estimation (MLE) is one of the most widely used methods for estimating the parameters of the Two-Sided Power Distribution (TSPD).

#### 2.2.1 Classical MLE approach.

Let $X_{\text{1}},X_{\text{2}}\cdots ,X_{n}$ be a sample from the TSPD with order statistics $X_{\left(\text{1}\right)}\leq X_{\left(\text{2}\right)}\leq \cdots \leq X_{\left(n\right)}$. Since the kernel of the TSPD is derived from the uniform distribution, the contribution of each observation is given by $X_{\left(j\right)}/\theta$ if $X_{\left(j\right)}\leq \theta$ and ${(\text{1}-X}_{\left(j\right)})/\left(\text{1}-\theta \right)$ if $X_{\left(j\right)}\geq \theta$.

For each r, the criterion function is defined as


$$M(r)=\prod_{J=\text{1}}^{r-\text{1}}\frac{X_{\left(j\right)}}{X_{\left(r\right)}}\prod_{j=r+\text{1}}^{n}\frac{{\text{1}-X}_{\left(j\right)}}{\text{1}-X_{\left(r\right)}}$$ (5)

The MLE estimates of the parameters are given as,


$$\hat{\theta }=X_{\left(\hat{r}\right)},\;\hat{\alpha }=\frac{-n}{lnM\left(\hat{r}\right)}$$ (6)

where $\hat{r}={argmax}_{\text{1}\leq r\leq s}M(r)$. Although this method is fast and computationally efficient, restricting θ to be an observed sample value can result in estimates that are sample-dependent and not smooth, thus limiting their generalizability.

#### 2.2.2 Continuous (Optimized) MLE approach.

To overcome the limitations of the classical MLE, we propose a more flexible and continuous MLE approach. In this formulation, the parameters θ and α are estimated over a continuous parameter space using numerical optimization. The log-likelihood function is defined as


$$logL(\theta ,\alpha )=\sum_{i=\text{1}}^{n}logf\left(X_{i}\;\;\right|\;\theta ,\alpha )$$ (7)

Here, $f\left(X_{i}\;\right|\;\theta ,\alpha )$ is the piecewise PDF defined in Equation (1). The log-likelihood function is maximized under the constraints θ∈(0, 1), and α>0, typically using numerical methods such as quasi-Newton algorithms or grid search techniques.

#### 2.2.3 Hybrid MLE approach.

In this study, we adopt a hybrid MLE strategy, which consists of two stages:

1. Initial Estimation: A fast and consistent initial estimate is obtained using Equation (6).
2. Continuous Optimization: This estimate serves as a starting point for optimizing the log-likelihood function defined in Equation (7) over the continuous domain.

This hybrid strategy offers both a fast and interpretable initialization and improved robustness through continuous refinement, resulting in more stable and generalizable parameter estimates.

### 2.3 Alternative derivation of the mean

While the classical expected value of the TSPD is available in closed form, it may not provide a good approximation of the sample mean when the sample size is small or the data is imbalanced. To overcome this issue, we propose an alternative mean formulation derived from the convex representation in Equation (4)


$$\mu^{*}=\text{w}\;\mu_{\text{1}}+(\text{1}-\text{w})\;\mu_{\text{2}}$$ (8)

where $\mu_{\text{1}}=E\left[(X\;|\;X\in [\text{0},\;\theta ]\right]=\alpha \theta /\left(\alpha +\text{1}\right)$ and $\mu_{\text{2}}=E\left[(X\;|\;X\in [\theta ,\;\text{1}]\right]=\left(\alpha \theta +\text{1}\right)/\left(\alpha +\text{1}\right)$.

This weighted formulation better approximates the empirical mean in small-sample scenarios, as demonstrated in the simulation experiments presented in the Results section.

### 2.4 Theoretical estimation of the probability Pr{Y<X}

For continuous random variables defined over the same compact interval, the probability Pr{Y<X} is a central metric, especially in reliability theory and stress-strength models. This probability can be expressed as


$$\kappa =\int_{\text{0}}^{\infty }F_{Y}(t)dF_{X}(t),\;X>\text{0},\;Y>\text{0}$$ (9)

This reliability-based probability has been extensively studied in the literature. Bhattacharyya and Johnson (1974) introduced a general reliability framework and computed κ in the context of order statistics and threshold-based criteria [7]. Dewanji and Rao (2001) further examined κ for various parametric models under stress-strength scenarios [8]. Gökdere and Gürcan (2015) evaluated κ using stratification techniques when X and Y followed exponential and Erlang distributions, thereby reducing computational complexity [11].

Van Dorp and Kotz (2002) extended this analysis to the two-sided power distribution (TSPD), deriving an analytical form of κ using the incomplete beta function when both X and Y are TSPD distributed [3]. Their work emphasized how shape and location parameters affect the reliability metric.

In this study, we consider $X\sim TSPD(\theta_{\text{1}},\;\alpha_{\text{1}})$ and $Y\sim TSPD(\theta_{\text{2}},\;\alpha_{\text{2}})$, where both are supported on [0, 1]. Due to the piecewise-defined nature of the two-sided power distribution, the integration domain is decomposed into nine subregions based on the positions of $\theta_{\text{1}}$ and $\theta_{\text{2}}$.

Let


$$l_{\text{1}}=\left\{\text{0}\leq Y\leq \theta_{\text{2}}\right\},\;l_{\text{2}}=\left\{\theta_{\text{2}}\leq Y\leq \theta_{\text{1}}\right\},\;l_{\text{3}}=\left\{\theta_{\text{1}}\leq Y\leq \text{1}\right\}$$

$$l_{\text{4}}=\left\{\text{0}\leq X\leq \theta_{\text{2}}\right\},\;l_{\text{5}}=\left\{\theta_{\text{2}}\leq X\leq \theta_{\text{1}}\right\},\;l_{\text{6}}=\left\{\theta_{\text{1}}\leq X\leq \text{1}\right\}$$

and define $(Y,X)\in l_{ij}=\left\{(Y,X):Y\in l_{i},\;X\in l_{j}\right\}$.

For the key subregions, the conditional probabilities are given as,


$$Pr\left\{Y<X|(Y,X)\in l_{\text{1}\text{4}}\right\}={\left(\theta_{\text{2}}/\theta_{\text{1}}\right)}^{\alpha_{\text{1}}}\frac{\alpha_{\text{1}}}{\alpha_{\text{1}}+\alpha_{\text{2}}}$$ (10)

$$Pr\left\{Y<X|(Y,X)\in l_{\text{2}\text{5}}\right\}=Betainc\left(\theta_{\text{1}},\alpha_{\text{1}},\alpha_{\text{2}}+\text{1}\right)-Betainc\left(\theta_{\text{2}},\alpha_{\text{1}},\alpha_{\text{2}}+\text{1}\right)$$ (11)

$$Pr\left\{Y<X|(Y,X)\in l_{\text{3}\text{6}}\right\}={\left\{\frac{\text{1}-\theta_{\text{1}}}{\text{1}-\theta_{\text{2}}}\right\}}^{\alpha_{\text{2}}}\frac{\alpha_{\text{1}}}{\alpha_{\text{1}}+\alpha_{\text{2}}}$$ (12)

Here, Betainc denotes the incomplete beta function. The respective probability masses of the subregions are given by


$$p_{\text{1}\text{4}}=Pr\left\{(Y,X)\in l_{\text{1}\text{4}}\right\}=\frac{\theta_{\text{1}}^{*}\theta_{\text{2}}^{*}\theta_{\text{2}}}{\theta_{\text{1}}},\;p_{\text{1}\text{5}}=Pr\left\{(Y,X)\in l_{\text{1}\text{5}}\right\}=\frac{\theta_{\text{1}}^{*}\theta_{\text{2}}^{*}({\theta_{\text{1}}-\theta }_{\text{2}})}{\theta_{\text{1}}}$$

$$p_{\text{1}\text{6}}=Pr\left\{(Y,X)\in l_{\text{1}\text{6}}\right\}=\left(\text{1}-\theta_{\text{1}}^{*}\right)\theta_{\text{2}}^{*},\;p_{\text{2}\text{5}}=Pr\left\{(Y,X)\in l_{\text{2}\text{5}}\right\}=\frac{\theta_{\text{1}}^{*}(\text{1}-\theta_{\text{2}}^{*})({\theta_{\text{1}}-\theta }_{\text{2}})}{(\text{1}-\theta_{\text{2}})\theta_{\text{1}}}$$

$$p_{\text{2}\text{6}}=Pr\left\{(Y,X)\in l_{\text{2}\text{6}}\right\}=\frac{{(\text{1}-\theta }_{\text{1}}^{*})(\text{1}-\theta_{\text{2}}^{*})({\theta_{\text{1}}-\theta }_{\text{2}})}{\left(\text{1}-\theta_{\text{2}}\right)}$$

$$p_{\text{3}\text{6}}=Pr\left\{(Y,X)\in l_{\text{3}\text{6}}\right\}=\frac{{(\text{1}-\theta }_{\text{1}}^{*})(\text{1}-\theta_{\text{2}}^{*})(\text{1}-\theta_{\text{1}})}{\left(\text{1}-\theta_{\text{2}}\right)}$$

Hence, the full expression for the estimated probability $\kappa^{*}$ is


$$\kappa^{*}=Pr\left\{Y<X|l_{\text{1}\text{4}}\right\}p_{\text{1}\text{4}}+Pr\left\{Y<X|l_{\text{2}\text{5}}\right\}p_{\text{2}\text{5}}+Pr\left\{Y<X|l_{\text{3}\text{6}}\right\}p_{\text{3}\text{6}}+p_{\text{1}\text{5}}+p_{\text{1}\text{6}}+p_{\text{2}\text{6}}$$ (13)

In the computation of the proposed probability estimate $\kappa^{*}$, the values $\theta_{\text{1}}^{*}=\theta_{\text{1}}$ and $\theta_{\text{2}}^{*}=\theta_{\text{2}}$ were adopted to ensure consistency with the assumptions used in the simulation experiments. This choice guarantees methodological coherence between the theoretical formulation and the empirical findings, thereby enabling a direct evaluation of $\kappa^{*}$ against the empirical benchmarks.

## 3. Results

To assess the effectiveness of the proposed method, a series of simulation studies was conducted under varying sample sizes and parameter configurations. Additionally, a real-world dataset was analyzed to validate the applicability of the approach.

### 3.1 Simulation study for the mean estimation

This section presents the simulation results conducted to assess the performance of the proposed convex mean estimator against the classical mean estimator in the context of the Two-Sided Power Distribution (TSPD). The key metrics evaluated include empirical mean, absolute bias, and mean squared error (MSE), computed across various sample sizes ranging from n=10 to n=200, based on 100 Monte Carlo replications per case. The corresponding simulation codes are provided as Supporting Information (S1 and S2 Text).

**Fig 1:**
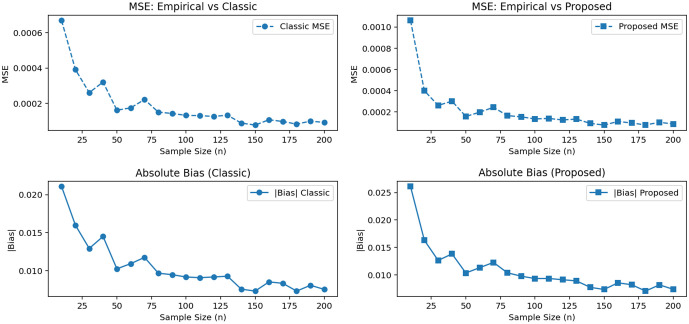
Mean Squared Error (MSE) and absolute bias of the classical and proposed mean estimators relative to the empirical sample mean for the TSPD (θ=0.7, α=1.5).

Fig 1 compares the MSE and absolute bias of the classical and proposed mean estimators for the TSPD with parameters θ=0.7 and α=1.5, each evaluated relative to the empirical sample mean. The classical estimator consistently exhibits low MSE across all sample sizes, reflecting its strong theoretical efficiency. When absolute bias is examined, the proposed estimator produces systematically smaller bias, particularly in small and moderate samples, while its MSE remains close to that of the classical estimator and decreases rapidly as n increases.

Taken together, these results reveal a clear bias–variance trade-off: the classical estimator offers slightly lower variance (lower MSE), whereas the proposed estimator provides improved bias performance without a meaningful loss in efficiency.

### 3.2 Simulation study for Pr{Y<X}

**Fig 2:**
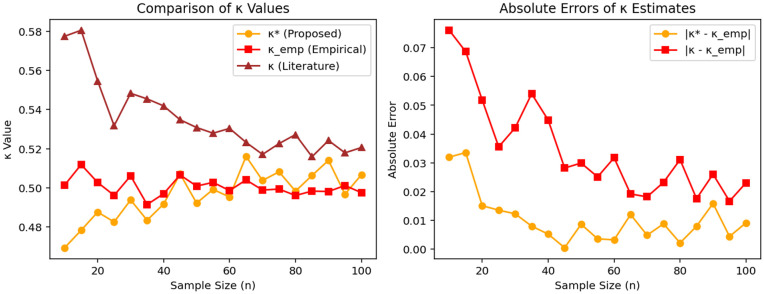
Estimated probabilities ${(\kappa }^{*})$, literature-based (κ), and empirical values ${(\kappa }_{Emp}$) across varying sample sizes (left), with corresponding absolute errors (right).

Fig 2 presents a comparative analysis of the proposed estimator $\kappa^{*}$, the traditional literature-based approximation κ, and the empirical values $\kappa_{Emp}$ across varying sample sizes.

In the left panel, the behavior of each estimator is examined as n increases. The proposed estimator $\kappa^{*}$ exhibits values that are consistently closer to the empirical estimates, particularly for small sample sizes (e.g., n≤40). As the sample size increases, both estimators tend to converge toward the empirical values; however, the trajectory of $\kappa^{*}$ is more stable and exhibits reduced deviations overall. This indicates that the proposed approach offers better adaptability and consistency, even under limited data scenarios.

The right panel shows the absolute bias of both estimators from the empirical values. These results clearly demonstrate that the proposed method maintains lower absolute errors across most sample sizes. Notably, the advantage is more pronounced in the small-sample regime, where accurate estimation is typically more difficult. The steadily decreasing trend in the error of $\kappa^{*}$ as n increases also reflects its favorable asymptotic behavior.

Overall, these findings suggest that the proposed estimator $\kappa^{*}$ serves as a more reliable and data-responsive alternative to traditional estimation techniques for Pr{Y<X}, particularly in applications involving small or moderate sample sizes, such as reliability analysis and clinical studies.

### 3.3 Real-world data application

In addition to the simulation-based evaluations, the proposed methodology was tested on a real-world dataset to assess its practical effectiveness. Specifically, a classical stress-strength dataset consisting of paired observations of load and strength was utilized. The dataset includes 20 matched observations obtained from publicly available reliability engineering records. The dataset corresponds to a classical example widely cited in the stress-strength reliability literature. Specifically, it originates from the failure data reported by Barlow and Campo (1975), which has been frequently referenced in system reliability studies [12].

Since the two-sided power distribution (TSPD) is defined on a bounded domain [0,1], both the Y and X values were linearly normalized to this interval before the analysis. The empirical probability $\kappa_{Emp}$ was computed directly from the sample, yielding a value of 0.90.

Parameter estimation for the two variables was then carried out using order-statistic-based moment-type estimators. The classical stress-strength reliability expression κ was derived based on the known closed-form expression from the literature for two TSPD variables with parameters $\left(\theta_{\text{1}},\;\alpha_{\text{1}}\right)$ and $\left(\theta_{\text{2}},\;\alpha_{\text{2}}\right)$. In parallel, the proposed estimator $\kappa^{*}$, derived using partitioned conditional probabilities and convex weighting (as given in Equation (13)), was also computed.

Table 1 presents the results obtained from this real dataset, comparing the empirical value $\kappa_{Emp}$ with the classical and proposed estimators in terms of bias and mean squared error (MSE).

**Table 1:** Estimation of Pr{Y < X} on a real dataset: comparison between the proposed and classical methods.

	$\kappa^{*}$	$\kappa_{𝐝𝐚𝐭𝐚}$	κ	Bias($\kappa_{Emp}-\kappa$)	Bias($\kappa_{Emp}-\kappa^{*}$)	MSE($\kappa_{Emp}-\kappa$)	MSE($\kappa_{Emp}-\kappa^{*}$)
Value	0.9000	0.9000	0.8765	0.0235	0.0000	0.000553	0.0000

Table 1 clearly demonstrates that the proposed estimator $\kappa^{*}$ coincides with the empirical probability, exhibiting both zero bias and zero MSE in this specific case. This result supports the earlier findings from simulation studies, which suggested that the proposed method is more robust and adaptable in small-sample scenarios.

By contrast, the classical estimator κ underestimates the empirical reliability probability, resulting in a non-negligible bias and error. These findings confirm that the convex partitioning framework and simplified integral formulation of the proposed method effectively capture reliability characteristics without requiring strong distributional assumptions or large sample sizes.

It is worth noting that although the classical formula is derived under theoretical assumptions and provides useful analytical insight, it may fail to capture empirical dynamics when sample sizes are limited or when parameters are not well-separated. In such cases, the proposed approach offers a viable and practically accurate alternative.

## 4. Conclusion and discussion

This study proposes an alternative approach aimed at obtaining reliable parameter estimates for the Two-Sided Power Distribution (TSPD) under small-sample conditions. By redefining the TSPD’s density function as a convex combination of two independent uniform components, the model enhances both its theoretical flexibility and computational tractability. This formulation preserves the asymmetric features of the classical TSPD while enabling parameter estimation over a continuous domain, thus yielding more balanced and stable outcomes.

In particular, a simplified and computationally efficient formula is introduced for the probability Pr{Y<X}, which is frequently used in fields such as reliability engineering, quality control, and risk assessment. Compared to the more complex formulations developed by [3,10], the proposed expression demonstrates superior stability in small-sample settings. Inspired by the subregion-based integration strategy of Gökdere and Gürcan (2015), the density structure of TSPD is partitioned into nine regions to form a more flexible estimation framework [11].

Simulation studies revealed that the proposed alternative mean formulation yields estimates that are closer to the empirical sample mean than the classical theoretical mean, especially under high-variance scenarios where α<2. In estimating Pr{Y<X}, the proposed method outperforms traditional closed-form approaches by offering lower bias and tighter variability across repeated samples. These findings indicate significant advantages in engineering applications involving limited data.

The real-data application supports these simulation-based conclusions, demonstrating that conventional estimation methods often produce biased results due to their sensitivity to underlying assumptions. In contrast, the proposed approach yields estimates that are both empirically consistent and robust, making it particularly useful for experimental studies with small sample sizes.

Compared to other flexible distributions in the literature—such as the beta, Kumaraswamy, or piecewise Weibull distributions—the proposed method offers advantages in terms of computational efficiency, theoretical clarity, and sensitivity to sample size [2,12]. Nonetheless, the current formulation’s applicability is restricted to the [0,1] interval. Future work could explore generalized variants of TSPD (e.g., shifted or scaled versions) to overcome this limitation. In addition, benchmarking against Bayesian or bootstrap-based methods would help assess the external validity and robustness of the proposed estimator.

Moreover, the proposed methodology can be directly integrated into stress–strength reliability models in applied engineering problems. Inference can be further strengthened by bootstrap-supported confidence intervals, which enhance robustness. Finally, comparative studies with other flexible distributions, such as the beta or Kumaraswamy distributions [13], represent promising avenues for future research.

In conclusion, this study enhances the theoretical and empirical estimation capacity of the TSPD, offering a more stable, computationally feasible, and practically relevant solution for small-sample scenarios.

## Supporting information

S1 Text Python code used for simulation of mean estimation. (TXT)

S2 Text Python code used for simulation of reliability. (TXT)

S1 File Excel file containing the real-world dataset used in the stress–strength application. (XLSX)
